# Electronic Support for Retrospective Analysis in the Field of Radiation Oncology: Proof of Principle Using an Example of Fractionated Stereotactic Radiotherapy of 251 Meningioma Patients

**DOI:** 10.3389/fonc.2017.00016

**Published:** 2017-02-09

**Authors:** Sandra Rutzner, Rainer Fietkau, Thomas Ganslandt, Hans-Ulrich Prokosch, Dorota Lubgan

**Affiliations:** ^1^Department of Radiation Oncology, Erlangen University Hospital, Erlangen, Germany; ^2^Chair of Medical Informatics, Friedrich-Alexander-University of Erlangen-Nuremberg, Erlangen, Germany

**Keywords:** clinical data warehouse, MOSAIQ^®^, routine clinical data, secondary use of data, data retrieval, stereotactic radiotherapy, meningioma

## Abstract

**Introduction:**

The purpose of this study is to verify the possible benefit of a clinical data warehouse (DWH) for retrospective analysis in the field of radiation oncology.

**Material and methods:**

We manually and electronically (using DWH) evaluated demographic, radiotherapy, and outcome data from 251 meningioma patients, who were irradiated from January 2002 to January 2015 at the Department of Radiation Oncology of the Erlangen University Hospital. Furthermore, we linked the Oncology Information System (OIS) MOSAIQ^®^ to the DWH in order to gain access to irradiation data. We compared the manual and electronic data retrieval method in terms of congruence of data, corresponding time, and personal requirements (physician, physicist, scientific associate).

**Results:**

The electronically supported data retrieval (DWH) showed an average of 93.9% correct data and significantly (*p* = 0.009) better result compared to manual data retrieval (91.2%). Utilizing a DWH enables the user to replace large amounts of manual activities (668 h), offers the ability to significantly reduce data collection time and labor demand (35 h), while simultaneously improving data quality. In our case, work time for manually data retrieval was 637 h for the scientific assistant, 26 h for the medical physicist, and 5 h for the physician (total 668 h).

**Conclusion:**

Our study shows that a DWH is particularly useful for retrospective analysis in the radiation oncology field. Routine clinical data for a large patient group can be provided ready for analysis to the scientist and data collection time can be significantly reduced. Furthermore, linking multiple data sources in a DWH offers the ability to improve data quality for retrospective analysis, and future research can be simplified.

## Introduction

Routinely documented clinical data are of great importance for patient care as well as for research purposes (1, 2).

So far, the retrospective analyses in medical research have been predominantly performed manually, meaning that clinical data are often transferred by hand from routine clinical reports into a separate research database (3) and stored in standard office tools (e.g., Microsoft Excel spreadsheets), which are not validated for clinical research. The continuously increasing expansion of electronic documentation in the clinical treatment process creates a large amount of various databases (4); thus, manual retrospective analysis is currently quite ambitious and time consuming.

In the field of radiation oncology, data sets are large and heterogeneous (5). Electronic information systems contain patients’ data for imaging in the Radiology Information System and Picture Archiving and Communication System, for irradiation in the Clinical Information System (CIS), e.g., Oncology Information System (OIS, MOSAIQ^®^) and data of the current course of the patients’ disease in the electronic health record (EHR, e.g., Soarian^®^ Clinicals).

With the increasing amount of patient information captured in EHRs and CISs, more opportunities should be established to facilitate clinical research by obtaining routine clinical data from distributed databases for secondary use, though providing access to routine clinical data for secondary use is challenging in practice (6). One of the greatest challenges in clinical research is to define and implement health data standards for integration between routinely used subsystems (7, 8). Medical data are frequently distributed across multiple electronical information systems of several departments in different forms of documentation styles (9). Although most university hospitals already implemented commercial hospital information systems and started to develop comprehensive EHRs, there is still a gap between clinical care and using this data for medical research that needs to be filled (10, 11). Recent studies have focused on providing routine clinical data for research purposes, e.g., by using a single-source tumor documentation or supporting systems for patient recruitment into clinical trials in the field of radiation oncology (12) and intensive care (13).

Data warehouses (DWHs) are central repositories of integrated data from one or more disparate sources. They store current and historical data and are used for creating analytical reports for knowledge workers throughout the enterprise (14). The purpose of this study is to verify the possible benefit of a DWH for retrospective analysis and reflect differences in manual and automated data retrieval.

Using meningioma patients as an example, we performed a therapy evaluation by utilizing an integrated electronic research database system DWH (clinical DWH) of the Erlangen University Hospital (UKER) to make routine radiotherapy data available from various operational subsystems. This is one of the largest populations of meningioma patients treated with stereotactic radiotherapy (SRT) in a single institution with a comprehensive database due to a high overall survival rate and a long observation period of meningioma patients after SRT.^1^

We manually and electronically collected basic information (patient characteristics), radiotherapy, and outcome data of 251 meningioma patients, who were irradiated from January 2002 to January 2015 at the Department of Radiation Oncology of the UKER (see text footnote 1). Currently, manual data collection represents the “gold standard.” In our study, we compared the results of both the electronic and manual data retrieval process and determined the congruence of data. Moreover, we measured the corresponding time requirements for both retrieval methods and the involvement of personnel (physician, physicist, scientific associate).

## Materials and Methods

### Environment

Erlangen University Hospital (UKER) is a tertiary care hospital that has 1.368 beds and combines 24 departments, 18 independent divisions, 7 institutes, and 25 interdisciplinary centers. In 2015, over 60,000 inpatient and nearly 475,000 outpatient cases were treated (15). At the Department of Radiation Oncology, 130–150 patients with many different tumor entities are irradiated daily. Approximately, 32 patients with meningioma are irradiated annually.

For our study, an agreement for the usage of routine clinical data was signed by those departments of the UKER that were involved in the patients’ treatment (Neurosurgery, Neurology, Neuropathology, and Radiology). These regulatory requirements and institutional policies need to be reconciled to use clinical routine data for clinical research activities.

### Principles of Radiotherapy of Intracranial Meningioma

During the past two decades, SRT has become increasingly well known as a treatment option for meningiomas (16, 17). Adjuvant SRT is offered to all grades II and III meningioma patients, whereas symptomatic grade I meningioma patients only received SRT after incomplete resection. Inoperable grade I (symptomatic only), grades II and III meningioma are treated with primary SRT. SRT was performed using the stereotactic radiosurgery system Novalis™ (BrainLAB, Feldkirchen, Germany). Patients were treated on consecutive workdays, with one fraction per day (see text footnote 1). SRT was mostly given in 28, 30, or 25 fractions to a median reference dose of 54.0 Gy.

### Scientific Objective of the Retrospective Analysis

Based on the example of 251 patients with 275 intracranial meningiomas treated between January 2002 and January 2015 with SRT at the department of Radiation Oncology of the UKER, we have illustrated the workflow of manual and electronical supported data retrieval for this analysis. For determination of efficacy of SRT on long-term outcome (e.g., overall survival, local control), the relevant parameters (age, gender, tumor localization, WHO grading, and current disorders after radiotherapy), data of the computed tomography (CT) or magnetic resonance (MR) imaging (to determine the tumor status after therapy), and temporal dose distribution [fractionation, target volume (PTV), dose distribution of risk organs] were evaluated.

### Workflow of Manual and Electronical Supported Data Collection for Retrospective Analysis

#### Manual Data Retrieval

For the purpose of retrospective analysis, the Department of Radiation Oncology begins with specifying the research question and defining the patient collective. Here, the patient collective was identified by multiple reference sources (e.g., outdated medical records and databases, institutional statistics) and manually summarized in a separate chart (Microsoft Excel 2010). All medical data in the routine CISs and necessary data elements for each patient were manually and separately noted in an electronic document using Microsoft Excel 2010. The systems used for manual analysis are listed in Table 1.

**Table 1:** **Summary of clinical information systems (CISs) for manual data retrieval to evaluate routine medical data for retrospective analysis of patients with meningioma treated with stereotactic radiotherapy**.

Clinical application	Data source	Description
SAP IS-H^®^	Patient administration	SAP^®^-based CIS for patient administration and documentation of diagnosis and procedure during the clinical treatment process

Soarian Clinicals^®^	Electronic health record	Web-based clinical workstation that offers health information in a digital form

Web-RIS^®^	Imaging	Web-based CIS that offers medical, administrative, and imaging data in the field of radiology

GTDS^®^	Medical record for tumor documentation	Supports clinical cancer registry and provides information about medical treatment and follow-up

MOSAIQ^®^	Radiation Oncology	Oncology information system for radiation treatment and the control of the respective linear accelerator. It is integrated with imaging, planning, and therapy systems and contains the planned and actual dispense treatment parameters

Pinnacle^3^^®^	Radiation Oncology	Treatment planning software for localization of tumor volumes and verification of the individual radiation treatment plan

I-Plan RT^®^	Radiation Oncology	Treatment planning software for localization of tumor volumes and verification of the individual radiation treatment plan

To evaluate the time required for manual data retrieval, we documented the time needed to collect all necessary data elements from clinical source systems and manually transcribed them into an Excel spreadsheet.

### Electronically Supported Data Retrieval

In order to simplify retrospective analysis, we decided to use a tool that obtains routine clinical data from multiple CISs for secondary use. Since 2003, the UKER provides the clinical DWH research platform to scientists for numerous analyses. It has the ability to combine data from multiple clinical source systems and to provide it to the hospital users. The DWH stores clinical and administrative data from 22 different data sources (e.g. Accounting, Pharmacy, Surgery, Anesthesia, Pathology, and Radiology). For transformation of routine clinical data, it utilizes the open enterprise-class platform Cognos Data Manager. The database language Structured Query Language (SQL) is used for defining data structures, editing, and querying the databases.

**Figure 1:**
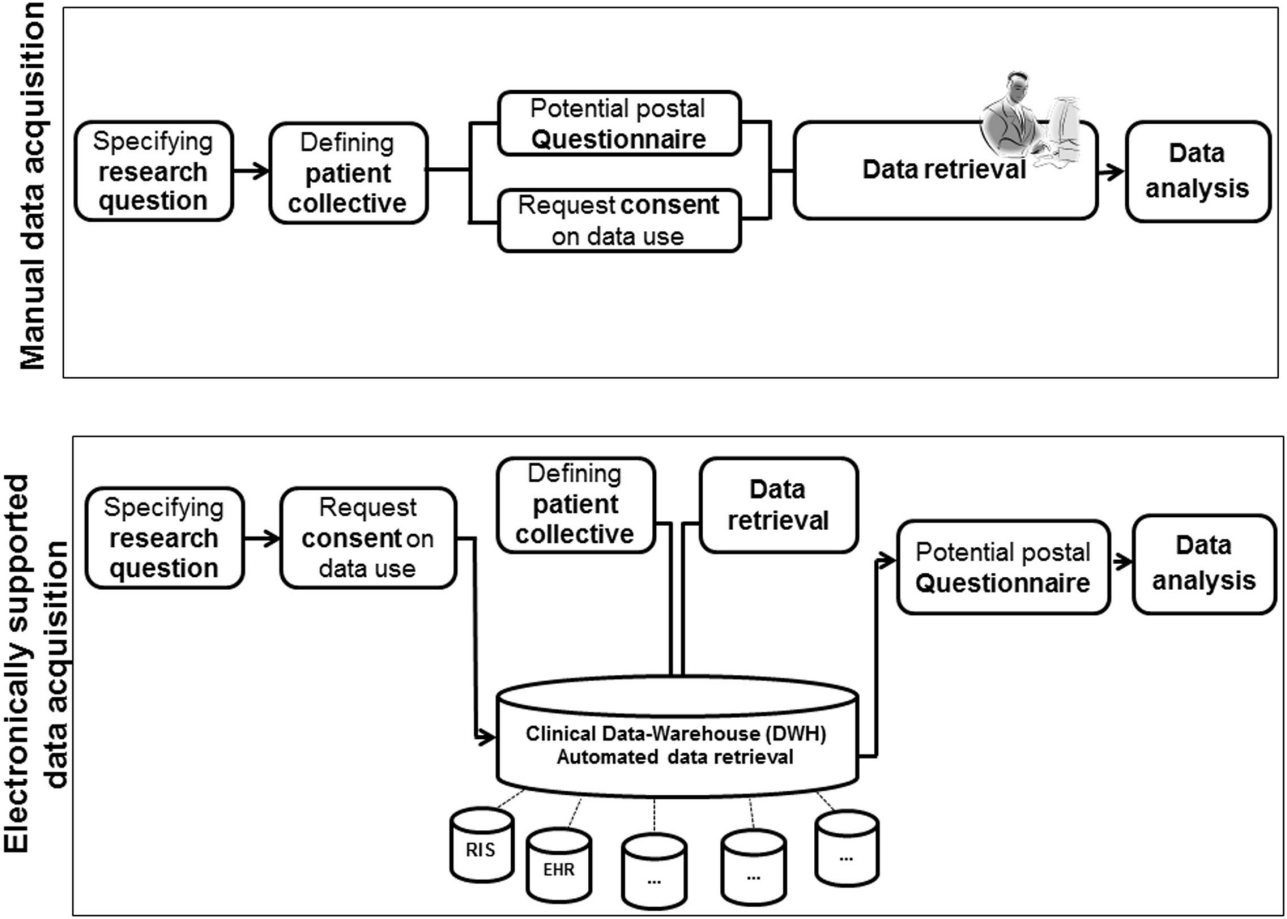
**Workflow of manually and electronically [data warehouse (DWH)] supported data retrieval for retrospective analysis**.

We used the DWH for defining a patient collective and obtaining routine clinical data from multiple CISs. The workflow of manual and electronical supported data retrieval for retrospective analysis is illustrated in Figure 1.

A database query based on routine clinical data from patient care was initiated to design a core data set for retrospective analysis (date of the last contact, date of the last imaging, life-status, beginning and end of the radiotherapy, fractionation, and dose). Selected data elements and the related data source system are shown in Table 2.

**Table 2:** **Overview of selected data elements and the related data source system for retrospective analysis**.

Data element	Description	Data type	Data source
Beginning of radiotherapy	First day of radiotherapy	Date [TT.MM.JJJJ]	SAP IS-H^®^

End of radiotherapy	Last day of radiotherapy	Date [TT.MM.JJJJ]	SAP IS-H^®^

Beginning of radiotherapy	First day of radiotherapy	Date [TT.MM.JJJJ]	MOSAIQ^®^

End of radiotherapy	Last day of radiotherapy	Date [TT.MM.JJJJ]	MOSAIQ^®^

Fractionation	Distribution of the total dose in separate doses	Numeric	MOSAIQ^®^

Dose	Dose value in Gy	Numeric [Gy]	MOSAIQ^®^

Date of last contact	Date of last contact/treatment at the Erlangen University Hospital (UKER)	Date [TT.MM.JJJJ]	SAP IS-H^®^

Date of the last imaging	Date of the last computed or magnetic resonance tomography of the brain	Date [TT.MM.JJJJ]	SAP IS-H^®^

Life-status	Date of death	Date [TT.MM.JJJJ]	Soarian Clinicals^®^

Actual patient contact details^a^	Master data of a patient (street, house number, postcode, and place of residence)	Free text	SAP IS-H^®^

Death due to tumor^a^	Death caused by tumor	Coded	GTDS^®^
		1 = yes	
		2 = no	
		999 = cannot be determined	

Tumor localization^a^	Localization of the tumor in the brain	Coded	Soarian Clinicals^®^
		1 = skull base	Web-RIS^®^
		2 = falx	
		3 = convexity	
		4 = meningeomatosis	

Histology/pathology^a^	Resection of the tumor enables the classification of the WHO-Grade (I,II,III)	Coded	Soarian Clinicals^®^
		1 = WHO I	
		2 = WHO II	
		3 = WHO III	
		999 = cannot be determined	

Previous radiotherapy^a^	Preceding radiation on the tumor area	Coded	Soarian Clinicals^®^
		1 = yes	SAP IS-H^®^
		2 = no	

Minimum/maximum dose^a^	Lowermost or paramount dose at radiation volume	Numeric [Gy]	i-Plan RT^®^, Pinnacle^3^^®^

PTV – volume^a^	Size of the planning target volume in cm^3^	Numeric [Gy]	i-Plan RT^®^, Pinnacle^3^^®^

Coverage PTV^a^	Proportion of the target volume within the reference isodose	Numeric [Gy]	i-Plan RT^®^, Pinnacle^3^^®^

Dose distribution on risk organs^a^	Tolerance dose on the critical organs (opticus right/left, chiasm, hippocampus)	Numeric [Gy]	i-Plan RT^®^, Pinnacle^3^^®^

*^a^Not included in the electronical [data warehouse (DWH)] analysis (currently not all listed data elements are accessible for the DWH or there were no suitable methods available for the extraction of the data elements)*.

The official system which was used for coding of the diagnosis is the 10th Revision of German Modification of the International Statistical Classification of Diseases (ICD-10) and for procedures the German “Operationen- und Prozedurenschlüssel Version 2015.”

Currently, not all listed data elements or source systems are accessible for the DWH (e.g., tumor as cause of death in the GDTS, the minimum or maximum dose, PTV-volume, coverage PTV, dose distribution on risk organs documented in the treatment planning software) or there were no suitable methods available for the extraction of the data elements (e.g., tumor localization, WHO grading, or several radiotherapy documented in Soarian^®^ Clinicals) at the time of analysis (Table 2). Therefore, they are not included in the electronical analysis.

### Integrating OIS MOSAIQ^®^ into the Clinical DWH of the UKER: Reusing Data from the OIS MOSAIQ^®^ for Retrospective Analysis

Since 2012, the Department of Radiation Oncology uses the OIS MOSAIQ^®^ developed by Elekta (Hamburg, Deutschland). It provides medical oncology data (e.g., demographic data, diagnoses, beginning and end of the radiotherapy, planned and administered fractionation and doses), regulates the respective linear accelerator, and is linked to imaging, planning, and therapy systems.

In order to make irradiation data available for retrospective analysis, we analyzed the table structure from the clinical system and transferred a copy of relevant data tables as read-only user during the non-productive clinical stage of radiotherapy (after 5 p.m.) into the staging area of the DWH. This process is called “*extraction*.” As a next step, we queried the DWH to select patients with a diagnosis of meningioma (ICD10-GM code D32.0, D32.9, C70.0, C70.9) and to identify the data elements *beginning and end of the radiotherapy, planned and administered fractionation and dose distribution*. Subsequently, we compared the results of the data base query and the manual data retrieval.

**Figure 2:**
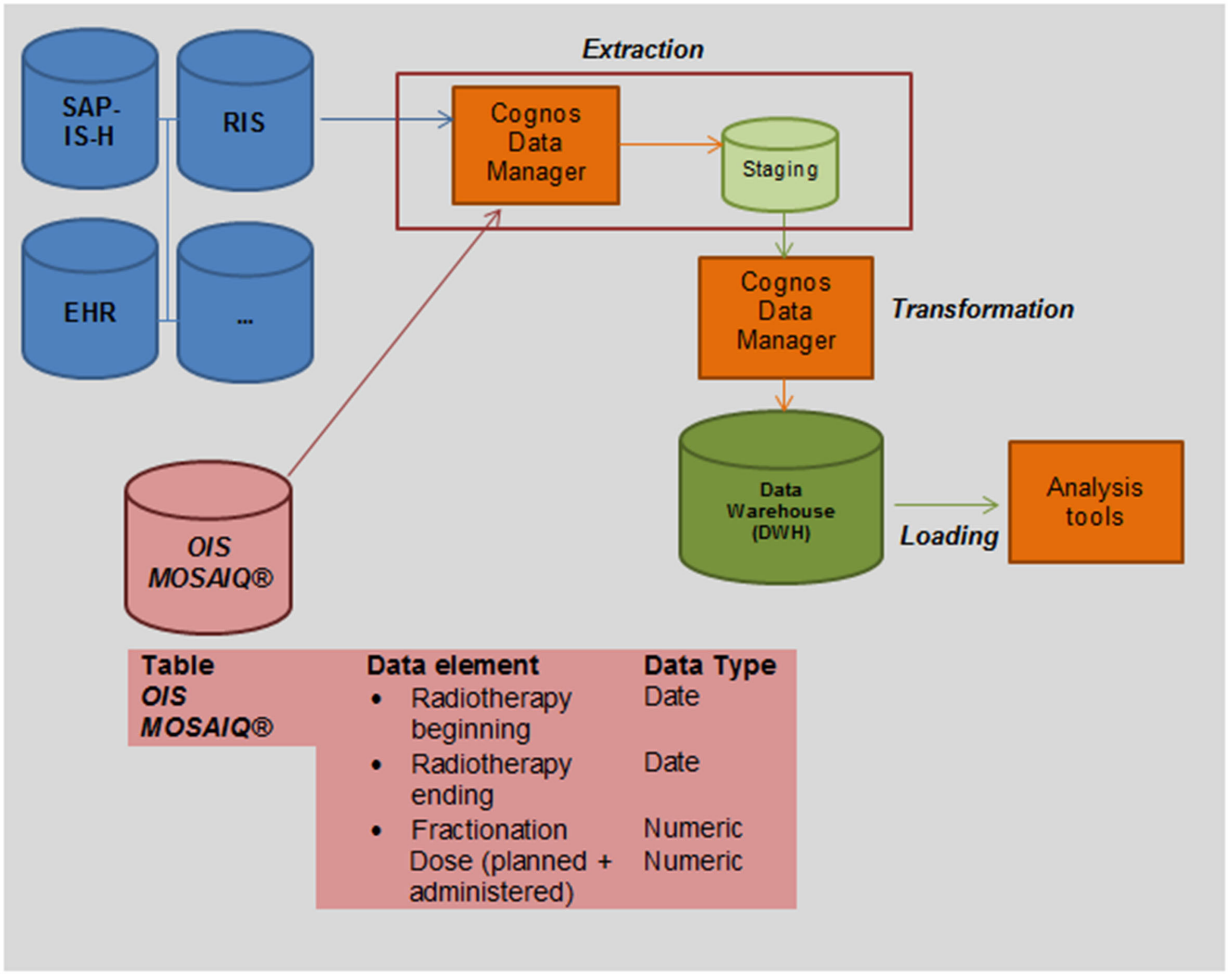
**Integrating Oncology Information System (OIS) MOSAIQ^®^ into the clinical data warehouse (DWH) of the UKER for secondary use: we transferred a copy of relevant data tables as read-only user during the non-productive clinical stage of radiotherapy (after 5 p.m.) into the staging area of the DWH (extraction)**. As a next step, we queried the DWH to identify relevant data elements (beginning and end of the radiotherapy, fractionation and dose).

In addition, unnecessary or inconsistent data can be corrected or extinguished at the staging area. This process is called “*transformation*.” The entire process is called *ETL* (*extraction, transformation, loading*) (18). The structure of the DWH and technical implementation of the clinical source system MOSAIQ^®^ is illustrated in Figure 2.

### Statistical Analysis and Ethics Committee Vote

Standard summary statistics and two-tailed 95% confidence intervals were calculated as appropriate. All statistical analyses were performed using the Statistical Package for the Social Sciences version 21 (IBM Corp., Armonk, NY, USA). The level of significance for all analyses was set at α = 0.05 (two-tailed).

Our institution obtained a positive ethics committee vote from the ethical review board for our research (reference number 347_16 Bc). All data used for the retrospective analysis was in anonymized form.

## Results

### Effectiveness of Patient Data Collection—DWH

A total amount of 275 data sets (case ID) from 251 (patients ID) patients were manually collected and stored in a Microsoft Excel spreadsheet. We counted 275 data sets (case ID) due to the fact that some patients had more than one lesion and thus were irradiated at multiple times.

Two hundred seventy-four electronic data sets (100%) from 250 patients were electronically collected because one patients’ data were not available for data protection reasons. The data congruence of the data elements “*beginning and end of the radiotherapy, date of the last contact, date of the last imaging and life-status (alive, dead)*,” were evaluated on the basis of manual data retrieval compared with the results of the DWH report.

### Manual Data Retrieval Compared with the Results of the DWH Report

The summary of selected data elements determined by manual and electronical supported data retrieval is shown in Table 3.

**Table 3:** **The comparison of manual data retrieval and the result of the data warehouse report**.

Data element	Data type	Data source *manual* correctness of the data (%)	Data source *electronical* correctness of the data (%)	Data source *MOSAIQ*^®^ correctness of the data (%)
Beginning of radiotherapy = first day of radiotherapy	Date [TT.MM.JJJJ]	252/274 (92.0)	257/274 (93.8)	

End of radiotherapy = last day of radiotherapy	Date [TT.MM.JJJJ]	252/274 (92.0)	257/274 (93.8)	

Beginning of radiotherapy = first day of radiotherapy	Date [TT.MM.JJJJ]	88/110 (80.0)		110/110 (100)

End of radiotherapy = last day of radiotherapy	Date [TT.MM.JJJJ]	88/110 (80.0)		110/110 (100)

Fractionation = distribution of the total dose in separate doses	Numeric	70/74 (94.6)		74/74 (100)

Dose = dispense dose value in Gy	Numeric [Gy]	70/74 (94.6)		74/74 (100)

Date of the last imaging = date of the last magnetic resonance or computed tomography imaging	Date [TT.MM.JJJJ]	248/274 (90.5)	236/274 (86.1)	

Date of last contact = date of the last contact/treatment at the UKER	Date [TT.MM.JJJJ]	232/274 (84.7)	274/274 (100)	

Life-status = date of death	Date [TT.MM.JJJJ] manual coded	14/14 (100)	7/14 (50.0)	
	0 = dead			
	1 = alive			

*χ^2^ test data source manual compared to data source electronical (MOSAIQ^®^ included) *p* = 0.009*.

*Data are number of data elements (%) unless otherwise stated. *p*-value: analysis of covariance, χ^2^ test in case of categorical data*.

### Data Element “Beginning of the Radiotherapy” and “End of Radiotherapy”

Two hundred fifty-two (92.0%) for manual and 257 (93.8%) for the electronical method out of 274 (100%) data elements “beginning of the radiotherapy” and “end of the radiotherapy” were identical. Thirty-nine (22 manual, 17 electronical) data elements were not identical.

Deviating results are more often generated by the manual than the electronical data retrieval method. Manual data retrieval produced 22/274 (8%) deviating results: this difference was caused by the fact that in 22 cases the treatment date of radiation was incorrectly documented in the discharge letter and the incorrect dates were transferred into the Microsoft Excel spreadsheet.

The DWH determined the correct treatment date for these 22 patients. However, the DWH query produced 17/274 (6.2%) deviating results due to an error in the data base query. The query was carried out patient-based (patients ID) instead of case-based (case ID). If a patient (patients ID) was treated multiple times over several years (case ID) only the latest “date of beginning and the end” was identified. For a flawless determination of the treatment (case ID), date the SQL statement of the data base query has to be adjusted for future data exports.

### Data Element “Date of the Last Imaging”

Of the 274, 248 (90.5%) by manual and 236 (86.1%) by electronical retrieval data elements were identical.

Differing results are more often generated by the electronical (38/274) than the manual (26/274) data retrieval method. Manual data retrieval produced 9.5% of inconsistent data: this difference was caused by the fact that over the course of time of manual data retrieval, an additional imaging was performed for 26 patients; thus, manually collected data were already outdated.

The DWH report determined 38 cases (13.9%) of diverging data: for 38 patients an imaging was performed at an external hospital. The information about external imaging is not accessible by a database query as it is based on the documented procedure code in the source system of the UKER.

### Data Element “Date of the Last Contact”

All data elements collected electronical were identical. Deviating results are only caused by the manual (42/274, 15.3%) data retrieval method. There were two reasons for this: first, for 18 patients the date of the last contact was incorrectly transferred from the source system into the Excel spreadsheet. Second, during the time of analysis, 24 patients were being treated again in another department at the UKER, and subsequently, manually collected data were already outdated.

### Data Element “Life-Status”

Overall 14 (5.6%) of 251 evaluated patients died. For all 14 patients, the day of death was manually collected. Seven (50.0% of all deceased) patients were overlooked by the DWH report because no information about their death was documented in the EHR (Soarian^®^ Clinicals) as the date of death is only documented for patients who died at the UKER.

### Effectiveness of Patient Data Collection—OIS

Fractionated SRT is documented in the OIS MOSAIQ^®^ since June 2012. We identified 110 suitable values for 74 patients (74 stereotactic irradiation + 36 data values for boost irradiation) since the system went into operation at the department of Radiation Oncology and transferred them into the DWH. We collected the data elements “beginning and end of radiotherapy, distributed dose and fractionation” by querying the DWH and compared the results with the manual data retrieval.

### Manual Data Retrieval Compared with the Results of the Mosaiq^®^ Report

#### Data Element “Beginning of the Radiotherapy” and “End of Radiotherapy”

Differing results were only caused by the manual data collection method (22/110): due to an incorrect date in the medical discharge letter manually retrieved data produced the deviating data for the beginning of radiotherapy and for the end of radiotherapy.

There were no deviating results by querying the source system MOSAIQ^®^ (DWH report) because the linear accelerator is regulated by the OIS that uses validation rules for data entry for every single fractionation in the primary source system.

### Data Element “Administered Dose and Fractionation”

In all, 94.6% (70/74) data elements were identical. The manual data retrieval methods lead to 4 (5.4%) deviating results because a medical physicist determined 4 false data elements of administered dose and fractionation on the basis of the paper-based health record, OIS MOSAIQ^®^ and the treatment planning systems I-plan RT^®^ or Pinnacle^3^^®^. There were no deviating results by querying the source system MOSAIQ^®^ (DWH report).

### Time Invested in Manual Data Retrieval

**Figure 3:**
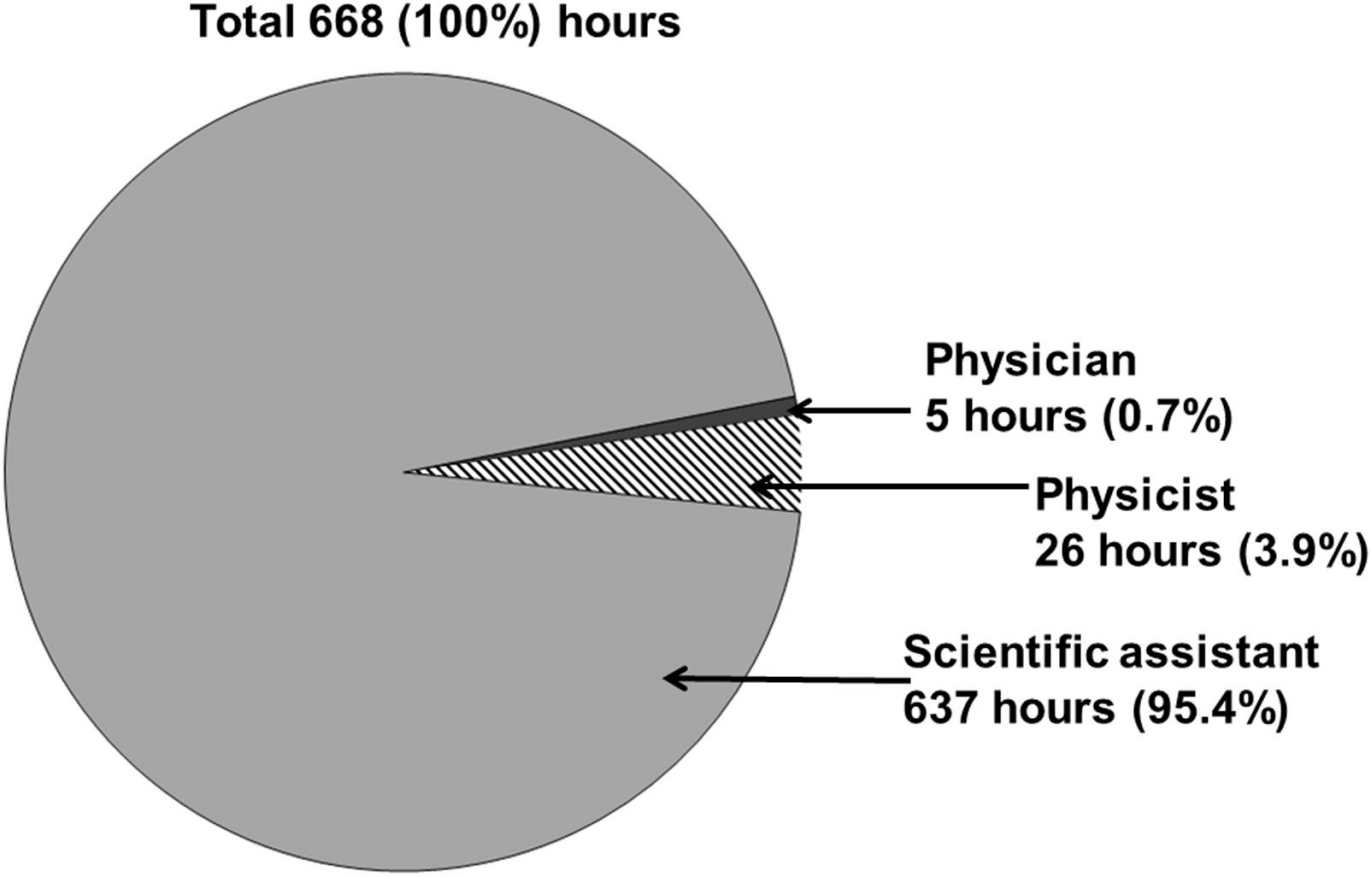
**The overall workload time of all involved professional groups for manually retrospective analysis of patients with meningioma treated with stereotactic radiotherapy is about 668 h**.

To evaluate the time required for manual data retrieval, we documented the time needed to collect all necessary data elements from clinical source systems and manually transmit them into a Microsoft Excel spreadsheet. The manual data retrieval required 668 h (Figure 3). The collection of all data elements took place over an extended period of time of about 24 weeks.

The scientific assistant required the largest amount of time while manually collecting routine clinical data in 637 h (95.4%) The support of a physician (5 h, 0.7%) and a medical physicist (26 h, 3.9%) was required (Figure 3). The physician analyzed actual MR or CT imaging (to determine localization, relapse, and progression of the tumor) and the medical physicist evaluated necessary data elements (PTV volume, fractionation, doses, minimum/maximum dose, coverage PTV, dose distribution of risk organs) on the basis of the paper-based health record and the treatment planning systems I-plan RT^®^ or Pinnacle^3^^®^.

### Time Consumption for Electronical Data Retrieval

In collaboration with a computer scientist of the Department of Medical Informatics and two scientific assistants of the Department of Radiation Oncology of the UKER, the DWH report was developed. Implementing the DWH query took 30 h that are composed of the definition, adjustment, and execution of the database query. For administrative activities (e.g., obtaining permission for data access by those departments of the UKER, which were involved in the patients’ treatment), we need additional 5 h.

The support of a medical physicist was not required to evaluate data elements (beginning and end of radiotherapy, administered fractionation, and dose) on the basis of the paper-based health record and the treatment planning systems I-plan RT^®^ or Pinnacle^3^^®^. For evaluating the data elements (PTV volume, minimum/maximum dose, coverage PTV, dose distribution of risk organs), the support of the medical physicist (approximately 20 h) and a physician (5 h) to analyze actual MR or CT imaging is still required.

## Discussion

The purpose of this study is to verify possible benefits of a clinical DWH for retrospective analysis in the field of radiation oncology.

We compared two different methods of collecting routine clinical data: manually and electronically using DHW for secondary use of the scientific retrospective analysis.

In summary, our results indicated that the electronically supported data retrieval (DWH) showed an average of 93.9% correct data and a significantly better (*p* = 0.009) result compared to manual data retrieval (91.2%). Using a research, database (DWH) replaces manual activities and offers the ability to significantly reduce data collection time and labor while improving data quality. However, data integrity depends on the quality of a structured routine clinical documentation as well as the system requirements to get access to medical data in the clinical source systems. Furthermore, expert knowledge for the transformation of routine clinical data is necessary in practice.

In our study, manual data retrieval needed significantly more overall workload time (668 h) of all involved professional groups compared to implementing the DWH query (30 h). We needed the support of a physician (5 h) to manually analyze CT or MR imaging and a medical physicist (26 h) for evaluating necessary irradiation data elements (fractionation, dose distribution, coverage/PTV volume, minimum/maximum dose, dose distribution at risk organs). Up to now, the support of a physician (5 h) to analyze actual MR or CT imaging is still required. In order to completely automate the assignment of the medical physicist for retrospective analysis (evaluating the data elements coverage/PTV-volume, minimum/maximum dose, dose distribution at risk organs), the departmental planning-systems I-plan^®^ RT and Pinnacle^3^^®^ need to be made accessible for the DWH.

In addition, the long period of time necessary for retrieving data manually produced outdated databases and caused errors when transmitting data into an electronic format such as Microsoft Excel, which became evident in some cases of our study. Furthermore, data retrieval errors can easily be introduced because medical record data are not guaranteed to be accurate (e.g., incorrectly documented treatment date of radiation in the discharge letter of radiotherapy) and depend on the care and knowledge level of the scientific assistant. A related study by Roelofs et al. (19) that examined the benefit of a clinical DWH combined with tools for extraction of relevant parameters data for a radiotherapy trial supports this point of view. A DWH is beneficial for data collection time in addition to offering the ability to improve data quality.

Besides of benefits of data collection times and improving data quality, the strength of a DWH its ability to combine data from multiple clinical source systems and make it easily accessible for researchers. Though, before using routine data for research purposes, it is important to carefully verify this data and determine data integrity. In this context, Galster (20) has reviewed existing barriers for reusing routine data, he came to the conclusion that clinical data are not available when or where it is needed, even though data is present, the usage of the existing source is prohibited or cannot be routinely used in its available form. In our study, there are regulatory requirements and institutional agreements that need to be reconciled from the departments of the UKER that are involved in the patients’ treatment in order to use clinical routine data for clinical research activities.

Next to the challenges of gaining access to multiple data sources, another major barrier for data reuse is the fact that routine data cannot be used in its available form. Usually, clinical data are distributed across several tables in a generic form with coded values (21). In our analysis, some data (e.g., tumor localization, histology/pathology) are semi-structured values (mostly free-text format) and therefore can’t be used for automatically analysis. The data recorded in structured fields are more readily to be extracted from an EHR than data that was recorded in free text notes. Therefore, expert knowledge for the transformation of this data is necessary, and the accuracy of database queries mainly depends on a specific SQL statement. In addition, EHR data are frequently recorded inconsistently in a variety of formats that are complex, inaccurate, and often incomplete (22). For our study, it is a necessary condition that medical data are recorded completely in a specific data schema in order to automatically capture as much information as possible for retrospective analysis.

Furthermore, EHRs often do not tell a complete patient story, whether it may be those of a single institution or those aggregated across institutions (23). An example for this problem in our study is the date of death that is only documented in the clinical source system (EHR) for patients who died at the UKER. Moreover, the information about an external imaging is not routinely documented in a coded form in the EHR and is therefore not accessible for database queries. Consequently, medical details from external sources (e.g., life status in the GTDS^®^, imaging at an external hospital) must be requested or made available for automated data abstraction. This would be worthwhile in order to determine a patients’ life status as an electronical life-status comparison with the residents’ registration offices is prohibited due to privacy policy since 2008 and an amendment to the Bavarian Cancer Registry is made for provision in 2016 (24). To keep the medical routine data up to date, we send a specially designed questionnaire to the patients in order to assess the health-related outcome that are completed by patients themselves.

Additionally, routine clinical documentation in the primary source systems affects the research outcome: data quality for retrospective analysis is only as good as the routine clinical documentation in the primary source systems e.g., EHR. Therefore, Kessel et al. (5) have developed a professional data-based documentation system for analysis purposes where information about radiation therapy, diagnostic images, and dose distributions has been imported into a web-based system. They showed that the central storage of data outside of EHR leads to benefits of digital management, data analysis, and reusability of the results. In this context, Kirrmann et al. (9) developed and described a flexible browser based reporting and visualization system for clinical and scientific use by linking web-services/MOSAIQ^®^, the physician letter system MEDATEC, and central server MiraPlus (laboratory, pathology and radiology). They reported that all relevant data were available at all times in a simple manner, which improved their effectiveness resulting in a considerable amount of time saving.

In this context, one benefit of our retrospective analysis was that the gain of access to radiotherapy data from the clinical source system MOSAIQ^®^. Besides the data sets “beginning and the end of radiotherapy” for evaluating treatment outcomes of patients with meningioma, we also extracted irradiation parameters “planned and effectively implemented fractionation and dosage distribution” from the existing primary source (OIS). Due to the fact that the linear accelerator and the OIS both use validation rules for data entry in the primary source system, original routine data are not subsequently changed. As we have shown in our analysis, using original and unprepared data leads to a higher percentage of accurate data.

A summary of described limitations and potential solutions using a DWH are shown in Table 4.

**Table 4:** **Limitations for using a data warehouse (DWH) for retrospective analysis in the radiation oncology field**.

Limitations	Example in our study	Potential solution	Benefit
Restricted data access	Regulatory requirements and institutional agreements need to be reconciled from those departments, which were involved in the patients’ treatment	Amendment of agreement between the departments of UKER about using clinical routine data for clinical research activities	Data integrity
Variety of data formats	Data cannot be routinely used in its available form: expert knowledge for the transformation of this data is necessary	Implementing data standards for the secondary use of health data to support clinical research	Availability of all data elements for retrospective analysis
Data quality of routine clinical documentation	Medical data generated in the clinical treatment process are not guaranteed to be accurate Electronic health records often do not tell a complete patient story: e.g., life-status (GTDS^®^), imaging (e.g., computed tomography/MRT) at an external hospital	Using original and unprepared data from primary source systems Making external source systems (e.g., GTDS^®^) accessible for the DWH Request external sources	Higher data quality Data integrity

Although only a selected data set of the evaluation of patients with meningioma was examined and not all data were directly available in a DWH, our present study highlights the benefit of electronical supported data retrieval for secondary use. Thus, our goal is to adapt our approach to other types of tumors in radiation oncology and extract more parameters from the existing routine care documentation systems.

## Conclusion

Our present study shows that a DWH is particularly beneficial for retrospective analysis in the field of radiation oncology. Routine clinical data for a large patient group can be provided ready for analysis to the scientific operator, and data collection time can be reduced significantly. Furthermore, using a DWH provides the ability to improve data quality for retrospective analysis; thus, future research can be simplified. However, expert knowledge for the transformation of routine clinical data is still necessary and the quality of a structured routine clinical documentation in the CISs as well as the system requirements allowing access to medical data also affect the outcome.

## Ethics Statement

This study was carried out in accordance with the recommendations of the ethical review board of the Friedrich-Alexander-University of Erlangen-Nuremberg (FAU) with written informed consent from all subjects. All subjects gave written informed consent in accordance with the Declaration of Helsinki. The protocol was approved by the ethical review board of the Friedrich-Alexander-University of Erlangen-Nuremberg (FAU) (reference number 347_16 Bc).

## Author Contributions

SR: conducted data analysis, described throughout the manuscript, and major contributor to the writing of the manuscript and literature search. RF: clinical oncologist and principal of the research organization, involved in the design of the study, and reviewed the manuscript. TG: made substantial contributions to the acquisition of data, developed the data warehouse report, and reviewed the manuscript. H-UP: was involved in the design of the study and reviewed the manuscript. DL: major contributor to the writing of the manuscript, supervised the study, and major contributor to organization of the data analysis and manuscript. All authors read and approved the manuscript.

## Conflict of Interest Statement

The authors declare that the research was conducted in the absence of any commercial or financial relationships that could be construed as a potential conflict of interest.

## Footnotes

^1^Lubgan D, Rutzner S, Semrau S, Lambrecht U, Roessler K, Buchfelder M, et al. Effective long-term local results and prognostic factors after fractionated stereotactic radiotherapy of 257 intracranial meningeoma. *J Neuro Oncol* (submitted for publication).

## References

[1] Dentler K ten Teije A de Keizer N Cornet R. Barriers to the reuse of routinely recorded clinical data: a field report. Stud Health Technol Inform (2013) 192:313–7. 10.3233/978-1-61499-289-9-313 23920567

[2] Hailemichael MA Marco-Ruiz L Bellika JG. Privacy-preserving statistical query and processing on distributed OpenEHR data. Stud Health Technol Inform (2015) 210:766–70. 10.3233/978-1-61499-512-8-766 25991257

[3] Rosolski T Hergert M Mauermann K Blomel D Präklinisches management von TIA/Insult/Blutung: Eine retrospektive Analyse von drei aufeinanderfolgenden Jahren. Der Notarzt (2003) 19(03):114–9. 10.1055/s-2003-39534

[4] Miriovsky BJ Shulman LN Abernethy AP. Importance of health information technology, electronic health records, and continuously aggregating data to comparative effectiveness research and learning health care. J Clin Oncol (2012) 30(34):4243–8. 10.1200/JCO.2012.42.8011 23071233

[5] Kessel K Habermehl D Bohn C Jäger A Floca R Zhang L Datenbankbasierte digitale retrospektive Auswertung von Patientenkollektiven in der Radioonkologie. Strahlentherapie und Onkologie (2012) 188(12):1119–24. 10.1007/s00066-012-0214-0 23108385

[6] Davies JM Gao W Sleeman KE Lindsey K Murtagh FE Teno JM Using routine data to improve palliative and end of life care. BMJ Support Palliat Care (2016) 6:257–62. 10.1136/bmjspcare-2015-000994 26928173 PMC5013160

[7] Hackl WO Ammenwerth E SPIRIT: systematic planning of intelligent reuse of integrated clinical routine data. A conceptual best-practice framework and procedure model. Methods Inf Med (2016) 55(2):114–24. 10.3414/ME15-01-0045 26769124

[8] Richesson RL Andrews JE Krischer JP. Use of SNOMED CT to represent clinical research data: a semantic characterization of data items on case report forms in vasculitis research. J Am Med Inform Assoc (2006) 13(5):536–46. 10.1197/jamia.M2093 16799121 PMC1561787

[9] Kirrmann S Gainey M Röhner F Hall M Bruggmoser G Schmucker M Visualization of data in radiotherapy using web services for optimization of workflow. Radiat Oncol (2015) 10(1):1. 10.1186/s13014-014-0322-3 25601225 PMC4307130

[10] Prokosch H-U Ganslandt T Perspectives for medical informatics – reusing the electronic medical record for clinical research. Methods Inf Med (2009) 48(1):38–44. 10.3414/ME9132 19151882

[11] Wagner S Beckmann MW Wullich B Seggewies C Ries M Burkle T Analysis and classification of oncology activities on the way to workflow based single source documentation in clinical information systems. BMC Med Inform Decis Mak (2015) 15:107. 10.1186/s12911-015-0231-x 26689422 PMC4687307

[12] Ries M Prokosch H-U Beckmann MW Bürkle T. Single-source tumor documentation-reusing oncology data for different purposes. Oncol Res Treatment (2013) 36(3):136–41. 10.1159/000348528 23486003

[13] Köpcke F Kraus S Scholler A Nau C Schüttler J Prokosch H-U Secondary use of routinely collected patient data in a clinical trial: an evaluation of the effects on patient recruitment and data acquisition. Int J Med Inform (2013) 82(3):185–92. 10.1016/j.ijmedinf.2012.11.008 23266063

[14] Jyoti B Munjal R Upasna S Investigation into data warehouse to meet the growing demand of information analysis. Int J Adv Res Comp Sci Manag Stud (2015) 3(8):45–8.

[15] Universitätsklinikum Erlangen (UKER). Universitätsklinikum Erlangen, Zahlen und Fakten. (2016). Available from: http://www.uk-erlangen.de/presse-und-oeffentlichkeit/zahlen-und-fakten/

[16] El-Khatib M El Majdoub F Hunsche S Hoevels M Kocher M Sturm V Stereotactic LINAC radiosurgery for the treatment of typical intracranial meningiomas. Efficacy and safety after a follow-up of over 12 years. Strahlenther Onkol (2015) 191(12):921–7. 10.1007/s00066-015-0880-9 26253788

[17] Maranzano E Draghini L Casale M Arcidiacono F Anselmo P Trippa F Long-term outcome of moderate hypofractionated stereotactic radiotherapy for meningiomas. Strahlenther Onkol (2015) 191(12):953–60. 10.1007/s00066-015-0915-2 26490452

[18] Mate S Kopcke F Toddenroth D Martin M Prokosch HU Burkle T Ontology-based data integration between clinical and research systems. PLoS One (2015) 10:e0116656. 10.1371/journal.pone.0122172 25588043 PMC4294641

[19] Roelofs E Persoon L Nijsten S Wiessler W Dekker A Lambin P. Benefits of a clinical data warehouse with data mining tools to collect data for a radiotherapy trial. Radiother Oncol (2013) 108(1):174–9. 10.1016/j.radonc.2012.09.019 23394741 PMC5119279

[20] Galster G Why is clinical information not reused. Stud Health Technol Inform (2012) 180:624–8. 10.3233/978-1-61499-101-4-624 22874266

[21] Johnson SB Chatziantoniou D. Extended SQL for manipulating clinical warehouse data. Proc AMIA Symp (1999):819–23. 10566474 PMC2232585

[22] Hripcsak G Albers DJ. Next-generation phenotyping of electronic health records. J Am Med Inform Assoc (2013) 20(1):117–21. 10.1136/amiajnl-2012-001145 22955496 PMC3555337

[23] Hersh WR Weiner MG Embi PJ Logan JR Payne PR Bernstam EV Caveats for the use of operational electronic health record data in comparative effectiveness research. Med Care (2013) 51(8):S30–7. 10.1097/MLR.0b013e31829b1dbd 23774517 PMC3748381

[24] Bayerischer Rechts- und Verwaltungsreport (BayRVR). Staatskanzlei: Kabinett beschließt Bayerisches Krebsregistergesetz. (2016). Available from: http://bayrvr.de/2016/05/10/staatskanzlei-kabinett-beschliesst-bayerisches-krebsregistergesetz/

